# Angiogenesis, Osseointegration, and Antibacterial Applications of Polyelectrolyte Multilayer Coatings Incorporated With Silver/Strontium Containing Mesoporous Bioactive Glass on 316L Stainless Steel

**DOI:** 10.3389/fbioe.2022.818137

**Published:** 2022-02-11

**Authors:** Yi-Jie Kuo, Chia-Hsien Chen, Pranjyan Dash, Yu-Chien Lin, Chih-Wei Hsu, Shao-Ju Shih, Ren-Jei Chung

**Affiliations:** ^1^ Department of Orthopedic Surgery, Wan Fang Hospital, Taipei Medical University, Taipei, Taiwan; ^2^ Department of Orthopedic Surgery, School of Medicine, College of Medicine, Taipei Medical University, Taipei, Taiwan; ^3^ Department of Orthopedic Surgery, Shuang Ho Hospital, Taipei Medical University, New Taipei City, Taiwan; ^4^ School of Biomedical Engineering, College of Biomedical Engineering, Taipei Medical University, Taipei, Taiwan; ^5^ Department of Chemical Engineering and Biotechnology, National Taipei University of Technology (Taipei Tech), Taipei, Taiwan; ^6^ Department of Materials, Imperial College London, London, United Kingdom; ^7^ Department of Materials Science and Engineering, National Taiwan University of Science and Technology, Taipei, Taiwan

**Keywords:** polyelectrolyte multilayer coating, mesoporous bioactive glass incorporated with silver and strontium, angiogenesis, osseointegration, antibacterial

## Abstract

The main causes for failure in implant surgery are prolonged exposure of implants or wound and tissue ischemia. Bacterial infection caused by the surrounding medical environment and equipment is also a major risk factor. The medical risk would be greatly reduced if we could develop an implant coating to guide tissue growth and promote antibacterial activity. Mesoporous bioactive glasses are mainly silicates with good osteoinductivity and have been used in medical dentistry and orthopedics for several decades. Strontium ions and silver ions could plausibly be incorporated into bioactive glass to achieve the required function. Strontium ions are trace elements in human bone that have been proposed to promote osseointegration and angiogenesis. Silver ions can cause bacterial apoptosis through surface charge imbalance after bonding to the cell membrane. In this study, functional polyelectrolyte multilayer (PEM) coatings were adhered to 316L stainless steel (SS) by spin coating. The multilayer film was composed of biocompatible and biodegradable collagen as a positively charged layer, γ-polyglutamic acid (γ-PGA) as a negatively charged layer. Chitosan was incorporated to the 11th positively charged layer as a stabilizing barrier. Spray pyrolysis prepared mesoporous bioactive glass incorporated with silver and strontium (AgSrMBG) was added to each negatively charged layer. The PEM/AgSrMBG coating was well hydrophilic with a contact angle of 37.09°, hardness of 0.29 ± 0.09 GPa, Young’s modulus of 5.35 ± 1.55 GPa, and roughness of 374.78 ± 22.27 nm, as observed through nano-indention and white light interferometry. The coating’s antibacterial activity was sustained for 1 month through the inhibition zone test, and was biocompatible with rat bone marrow mesenchymal stem cells (rBMSCs) and human umbilical vein endothelial cells (HUVECs), as observed in the MTT assay. There was more hydroxyapatite precipitation on the PEM/AgSrMBG surface after being soaked in simulated body fluid (SBF), as observed by scanning electron microscopy (SEM) and X-ray diffraction (XRD). In both *in vitro* and *in vivo* tests, the PEM/AgSrMBG coating promoted angiogenesis, osseointegration, and antibacterial activity due to the sustained release of silver and strontium ions.

## Introduction

Surgical site infection is a common cause of surgical failure and occurs when pathogens proliferate at the site of a surgical incision or post-operative wound, and this also includes infections from the medical environment through the implant. According to the 2016 World Health Organization (WHO) report, surgical site infection occurs in up to 4.5% of all surgeries in some Nordic countries (World Health Organization, 2016). The Taiwan Centers for disease Control found that surgical site infection accounts for 5–6% of all medical care infections.

Although preventive measures have improved significantly in medical treatment, surgical site infection is still a major cause of surgical failure. Research indicates that there is a 1–2% incidence of infection by primary arthroplasty (Kurtz et al., 2012). Furthermore, the risk of re-infection is around 8% after revision surgery for prosthetic hips (Kunutsor et al., 2015) and knees (Kunutsor et al., 2016), though some studies have indicated values as high as 57.1% (Marculescu et al., 2006; Lee et al., 2010). These infections commonly occur due to a foreign body response between the wound and the surface of the material, which results in inadequate wound healing, repeated exposure to bacterial infection, and sensitivity to other related factors that can lead to implant failure (Esposito et al., 1998; Seebach and Kubatzky, 2019). Modern research has been focused on modifying the implant surface to effectively improve the response of the body and elongate the service lifetime. Metallic implants were some of the earliest biomaterials (Zreiqat et al., 2002; Chen and Thouas, 2015) and included 316L stainless steel (SS), titanium alloy, and nickel-titanium memory alloy. The surfaces of these materials can be modified to incorporate beneficial properties (Hanawa, 1999) such as chemical stability, biocompatibility, and controllable mechanical properties similar to those of human bones (Niinomi, 2002; Liu et al., 2014; Liu et al., 2015).

Osseointegration was first applied in 1962 with the definition as direct contact between implants and bone under the resolution level of the light microscope (Albrektsson and Wennerberg, 2019). These implants can only be fabricated with materials having osseointegrative properties. The ultimate aim of the implant is to be incorporated into the living tissue and substitute tissue functions (Guglielmotti et al., 2019). The success of osseointegration depends on the adhesion and healing ability of the bone cells and the implant. The prolonged recovery requires initial stabilization followed by second-stage healing until the bone cells are successfully calcified and tightly embedded in the surface of the implant and able to withstand internal and external forces (Legeros and Craig, 1993). The surface of osseointegrated implants can be modified by coating, anodizing, plasma treatment, sandblasting, and acid etching (Le Guehennec et al., 2007).

Polyelectrolyte multilayer (PEM) coating is a surface modification technique that is commonly used for implant modification, such as improving antibacterial properties, cell adhesion, antiadhesion, and drug delivery (De Avila et al., 2019; Jung et al., 2019; Kruk et al., 2019; Martins et al., 2020). PEM coatings are self-assembled and attached to implants using electrostatic attraction by stacking positively and negatively charged layers (Panda et al., 2021a). Electrostatic attraction can stabilize the coating layers and the interface between the substrates. PEM coating has significant benefits for implant surface modification, including adjustable mechanical properties (Zankovych et al., 2013), excellent biocompatibility (Hartmann and Krastev, 2017), and sufficient storage space for dopants to accommodate multiple functional layers. The coating can also be incorporated with a drug release agent such as mesoporous bioactive glass (MBG) that enables short to long-term release of antibacterial agents (Liu et al., 2018; Liu X et al., 2020).

Collagen is a natural abundant protein macromolecule present in bone, connective tissue, tendon, skin, and blood vessels that is consisting around 30% of total protein of human body (Liu et al., 2021). Due to enhance biocompatibility, intense mechanical properties, biodegradability, antibacterial properties, bone regeneration, initiating bio mineralization behaviors, crosslinking controllable process and cell adhesion, growth, it has been utilized in various biomedical application as implant materials including tissue remodeling, bone tissue engineering, bone reconstruction and wound healing process (Muthukumar et al., 2016; Chandran et al., 2019; Lin and Chiu, 2021; Wang et al., 2021; Yang et al., 2021). γ-polyglutamic acid (γ-PGA) is a synthetic polypeptide consisting of D- and L-form of naturally occurring glutamic acids which are connected through amide bonds between the α-amine and γ-carboxyl acidic groups (Panda et al., 2021). It has been utilized in several applications including tissue engineering, drug delivery, antibacterial studies, food purposes, and pharmaceutical industries, due to its high hydrophilicity, good biodegradability, biocompatibility, nontoxicity, edible nature, good cytocompatibility, and immunogenicity (Tsao et al., 2010; Panda et al., 2021a; Antunes et al., 2011; Khalil et al., 2016; Yang et al., 2021). Chitosan is a natural polysaccharide consisted of poly-β (1–4)-d-glucosamine obtained from chitin through alkaline deacetylation, that is found in crustacean shells (Panda et al., 2021b). It has been used in various applications, including drug delivery, tissue engineering, antibacterial and antifungal treatment due to its biodegradability, nontoxicity, biocompatibility, biological renewability, and non-antigenicity (Panda et al., 2019; Martins et al., 2020; Panda et al., 2021a; Panda et al., 2021b). The combination of Collagen and γ-PGA can intensify biomedical applications based on implant biomaterials due to their biodegradability, biocompatibility, nontoxicity, antibacterial properties, cell adhesion growth ability and bone remodeling behavior.

MBG has a large biocompatible surface area with excellent bioactivity and is popularly used as a drug carrier in clinical applications (Hoppe et al., 2011). Previous studies have loaded MBG with copper, silver (Ag), and graphene oxide as antibacterial agents. These materials release ions to bond with the cell membrane, causing membrane rupture and cell apoptosis due to surface charge imbalance (Asharani et al., 2009; Mcshan et al., 2014; Shih et al., 2017). In osseointegrated applications, low concentrations of strontium (Sr) ions can induce osteocalcin, bone sialoprotein, and osteoblast proliferation with high expression of alkaline phosphatase (ALP) to avoid osteoporosis. Sr ions can effectively increase bone formation and improve bone mineral density and structural properties in skull reconstruction tests (Dahl et al., 2001; Marie et al., 2001; Pilmane et al., 2017). Therefore, Ag and Sr-doped MBG (AgSrMBG) can release Ag and Sr ions simultaneously to inhibit bacterial proliferation and stimulate osteoblast mineralization and angiogenesis (Shi et al., 2015; Mao et al., 2017).

In this study, a multifunctional PEM coating was designed to inhibit bacterial activity, improve angiogenesis, promote osteoblast differentiation, and sustain long-term drug release. The multifunctional PEM coating was prepared on a 316L stainless steel (SS) substrate. The PEM coating is self-assembled through electrostatic attraction between 20 alternating layers of collagen with positive charge and γ-polyglutamic acid (γ-PGA) with negative charge, respectively. Each negatively charged layer contained AgSrMBG, and at 11th chitosan was used as a stabilizing layer owing to its excellent insulating properties. Multifunctional PEMs were analyzed *in vitro* for suitability in a complex biological environment as well as to evaluate their antibacterial properties in a cocultured medium with *E. coli*. Cell proliferation, mineralization, and biocompatibility were tested by MTT assay using mouse osteoblastic cell line MC3T3-E1 (acquired from American Type Culture Collection, ATCC CRL-2593), rat bone marrow mesenchymal stem cells (rBMSCs, primary culture) and human umbilical vein endothelial cells (HUVECs, ATCC CRL-1730). An *in vivo* rat skull defect test was used to observe angiogenesis and new bone formation.

## Materials and Methods

### Materials

Type I Collagen was provided from Victory Biotech Co. Ltd. (Taiwan); γ-PGA was purchased from Vedan Enterprise Corporation (Taiwan). Chitosan was obtained from Charming & Beauty Co. Ltd., (Taiwan). Calcium nitrate tetrahydrate (Ca(NO_3_)_2_. 4H_2_O), and tetraethyl orthosilicate (Si(OC_2_H_5_)_4_)) were purchased from Showa (Japan). Triethyl phosphate (C_2_H_5_)_3_PO_4_) and silver nitrate (AgNO_3_) were obtained from Alfa Aesar (United States). Tetracycline, 3-(4,5-dimethylthiazol-2-yl)-2,5-diphenyl tetrazolium bromide (MTT) and dimethyl sulfoxide (DMSO) were bought from Sigma-Aldrich (United States). Minimal essential medium alpha (α-MEM), fetal bovine serum (FBS), and phosphate-buffered saline (PBS) were purchased from Gibco (Thermo Fisher Scientific Corporation, United States). 4′,6-diamidino-2-phenylindole (DAPI) was obtained from Sigma-Aldrich (United States). Other chemicals were provided from a registered vendor and used directly.

### Preparation of SrMBG and AgSrMBG Powders

SrMBG and AgSrMBG powders were prepared using a spray pyrolysis (SP) technique. The MBG precursor was composed of 6.70 g tetraethyl orthosilicate, 1.40 g calcium nitrate tetrahydrate, 0.73 g triethyl phosphate, 6.95 g F127 surfactant, 40 ml of ethanol, and 1.00 g 0.5 M HCl, mixed at room temperature for 24 h. A second precursor solution for SrMBG consisted of 0.89 g strontium nitrate mixed in 180 ml distilled water for 1 h; and additional 0.12 g silver nitrate for AgSrMBG. Both precursors were then mixed for 1 h. The SrMBG or AgSrMBG precursor solutions were passed through three processing zones of SP: preheating, calcination, and pyrolysis at 400°C, 700°C, and 500°C, respectively. The SrMBG and AgSrMBG powders were collected using an electrostatic precipitator (Shih et al., 2016). The final SrMBG contained 10 wt% Sr; and AgSrMBG contained 10 wt% Sr and 1.64 wt% Ag.

### Pre-Treatment of Substrates

We used 316L SS as a substrate for the PEM coating. The substrate was pretreated with 100 ml of 5 M NaOH solution at 60°C for 3 h and washed three times with secondary water to obtain a negatively charged surface.

### Preparation of Film Forming Solutions

Collagen was used to serve as the positively charged layer in the PEM film. Collagen fiber (500 mg) was dissolved in 100 ml of UV-sterilized 0.5 N acetic acid solution in a UV-sterilized 500 ml serum bottle for 5 days. The negatively charged layer was prepared by dissolving 500 mg γ-PGA in 100 ml of sterilized secondary water and mixed in a 500 ml UV-sterilized serum bottle. Chitosan was added as a stabilizing layer in the PEM, and prepared by dissolving 500 mg of chitosan in 100 ml of sterilized secondary water and mixed with 200 μL of 0.5 M acetic acid in a 500 ml UV-sterilized serum bottle. To obtain a final PEM coating of 0.625 mg/ml SrMBG or AgSrMBG, 6.25 g of powders were mixed with 10 ml of γ-PGA solution in a 15 ml centrifuge tube. The mixed solution was sonicated and vortexed for 10 min.

### Formation of Polyelectrolyte Multilayer Films

Each respective layer of collagen, SrMBG (AgSrMBG)/γ-PGA, and chitosan was used spin-coated process to synthesis PEM films. 100 μL of the relevant solution was deposited on substrate surface, and spun at 2000 rpm for 5 s followed by at 4,000 rpm for 5 s. The final PEM coating was composed of up to 20 alternating layers of positively charged collagen and negatively charged γ-PGA containing SrMBG or AgSrMBG, with chitosan incorporated with the 11th stabilizing layer. The corresponding PEM films, SrMBG or AgSrMBG onto the PEM films were designated as (Collagen/γ-PGA)_n_, PEM/SrMBG and PEM/AgSrMBG respectively in which ‘n’ is the number of bilayers.

### Characterization of Coatings

We used X-ray diffraction (XRD, Malvern panalytical system (Netherlands) and scanning electron microscopy (SEM, Hitachi s-3000 N, Japan) to characterize the crystal structure and surface morphologies of samples before and after treatment with simulated body fluid (SBF). Contact angle measurement (DSA325, Kruss Advance, Japan), nanoindentation (TI 700 Ubi, Hysitron, United States), and white light interference microscopy were used to describe the surface properties, including surface hydrophobicity, mechanical properties, and layer thickness, of the PEM/SrMBG and PEM/AgSrMBG respectively. UV-vis spectroscopy (UV-vis, JASCO, V-650, Japan) was used to directly evaluate *E. coli* response to PEM in the MTT assay as well as to measure ALP and calcium (Ca) concentrations. Inductively coupled plasma-optical emission spectrometry (ICP-OES) was used to confirm the dopant concentrations in the MBG.

### Preparation of Antibacterial Test

Antibacterial activity was assessed using the inhibition zone test as per our previous study (Liu X. et al., 2020)*.* In this analysis, we used *E. coli* DH5α as the control bacteria. Briefly, we prepared (1 × 1) cm^2^ PEM samples loaded with SrMBG or AgSrMBG. Each sample was UV sterilized for 15 min. The sterilized samples were then co-cultured with *E. coli* DH5α, and inhibition zones were measured as the antimicrobial potency of samples and reported in centimeter (cm) at 1, 3, and 5 days.

### Preparation of *In Vitro* Test

We conducted *in vitro* tests to determine the suitability and biocompatibility of the coatings. Those assessments included PEM coating degradation, ion release, cell attachment, cell proliferation, cytotoxicity, ALP, and Ca concentration. We recorded the degradation of the coatings in a phosphate buffer saline (PBS) up to 90 days. This solution was also used to perform ICP-OES in evaluating ion release. Cell attachment and proliferation were assessed using 4′,6-diamidino-2-phenylindole (DAPI) fluorescent DNA staining, which binds to the adenine-thymine rich regions of the cell’s DNA. The maximum absorption wavelength of DAPI is 358 nm, and its emission maximum is at 461 nm. Fluorescence was used to estimate the number of cells and fluorescent images were used to observe cell attachment ability. Bioactivity was assessed by hydroxyapatite (HAp) formation, as observed by SEM, after treating the samples with 2 times the concentration of SBF up to 21 days. Crystal structure was characterized by XRD (Dash et al., 2020). Cytotoxicity tests were performed using the MTT assay as per our previous studies (Liu X et al., 2020)*.* To optimize concentrations to be used in the PEM films, cytotoxicity tests were performed by co-cultured osteoblastic cell line MC3T3-E1 with various concentrations of SrMBG and AgSrMBG. In this test, the MC3T3-E1, rBMSCs or HUVECs cells were seeded in each well-plated for 24 h to became adhere the surface. After that, samples were placed in each well plate and added medium (0.5 ml) with different concentrations, medium was changing in every 3 days and washed with PBS. After that, the MTT reagent was co-cultured with rBMSCs or HUVECs along with samples was placed in incubator under dark condition for 3 h, then formazan crystals were formed and dissolved with 1 ml of DMSO for 15 min with shaking. The cytotoxicity results were recorded as optical density (OD) values by UV-vis spectrophotometer at 570 nm on days 7, 14 and 21. ALP test was followed with our previous studies (Liu X et al., 2020). ALP concentration is an index of mature osteoblasts and is used to determine osteoblast proliferation associated with the material. In this test, rBMSCs were co-cultured with PEMs for 7, 14, and 21 days and mixed with 500 μL of 0.1% Triton x-100/0.1 M Tris buffer for 10 min. We mixed 50 μL of this solution with 200 μL of substrate buffer for 60 min and terminated the reaction with 100 μL of 1 M NaOH. The ALP results were recorded as OD values using a 405 nm UV-vis spectrophotometer. Calcium (Ca) content test was followed with our previous studies (Liu et al., 2020). In this test, the Ca concentration test is an index of cell osteogenesis, which occurs by cell-secreted ALP reacting with MBG. PEMs were co-cultured with rBMSCs for 0, 7, 14, and 21 days. The culture medium was replaced every 3 days. The samples were then washed three times with 1 ml of PBS and treated with 1 ml of 0.5 N acetic acid for 24 h. Finally, 10 μL of the sample solution was collected and mixed with 300 μL of *o*-cresolphtalein complexone (OCPC) reagent for 10 min to measure the Ca ion concentration by UV-vis absorption at 575 nm.

### Preparation of *In Vivo* Test

*In vivo* studies on 4-week-old Sprague-Dawley rats (average weight = 75–95 g) were performed to study the biocompatibility of the samples. The materials were surgically implanted into the skull defects of rats. We carried out the experiments at MacKay Memorial Hospital (Taipei, Taiwan) with regarding to the guidelines for the care and use of animals, which approved by the Affiliated Institutional Animal Care and Use Committee (IACUC) with the affidavit (MMH-A-S-105–24). At least 1 week prior to the experiment, the animals were well acclimatized and kept. Anesthesia was induced by intraperitoneal injection using a 0.1 ml Zoletil 50 and 0.2 ml Rompun 20 mix. A 6 mm diameter drill was used to puncture the top of the skull, where the packed MBG powder disc or PEM sample of 6 mm diameter and 1 mm thickness was fixed. After implantation, we analyzed the angiogenic and osteogenic capacities of the SrMBG, AgSrMBG, PEM/SrMBG and PEM/AgSrMBG. Histological analyses of hematoxylin and eosin (H&E) staining were conducted after one or 3 weeks.

### Statistical Analysis

All data were evaluated as the mean ± standard deviation (SD) with five replicates (*n* = 5). We used Statistical Package for Social Sciences (SPSS) (version 17.0.1) (Norusis/SPSS Inc., Chicago, IL) software to statistically analyze the results. Analysis of variance (ANOVA) was calculated to evaluate the significance data. The Bonferroni correction was used for data with statistically significant differences (*p* < 0.05).

## Results and Dicussion

### Optimization of Polyelectrolyte Multilayer/AgSrMBG Composite Films

**FIGURE 1:**
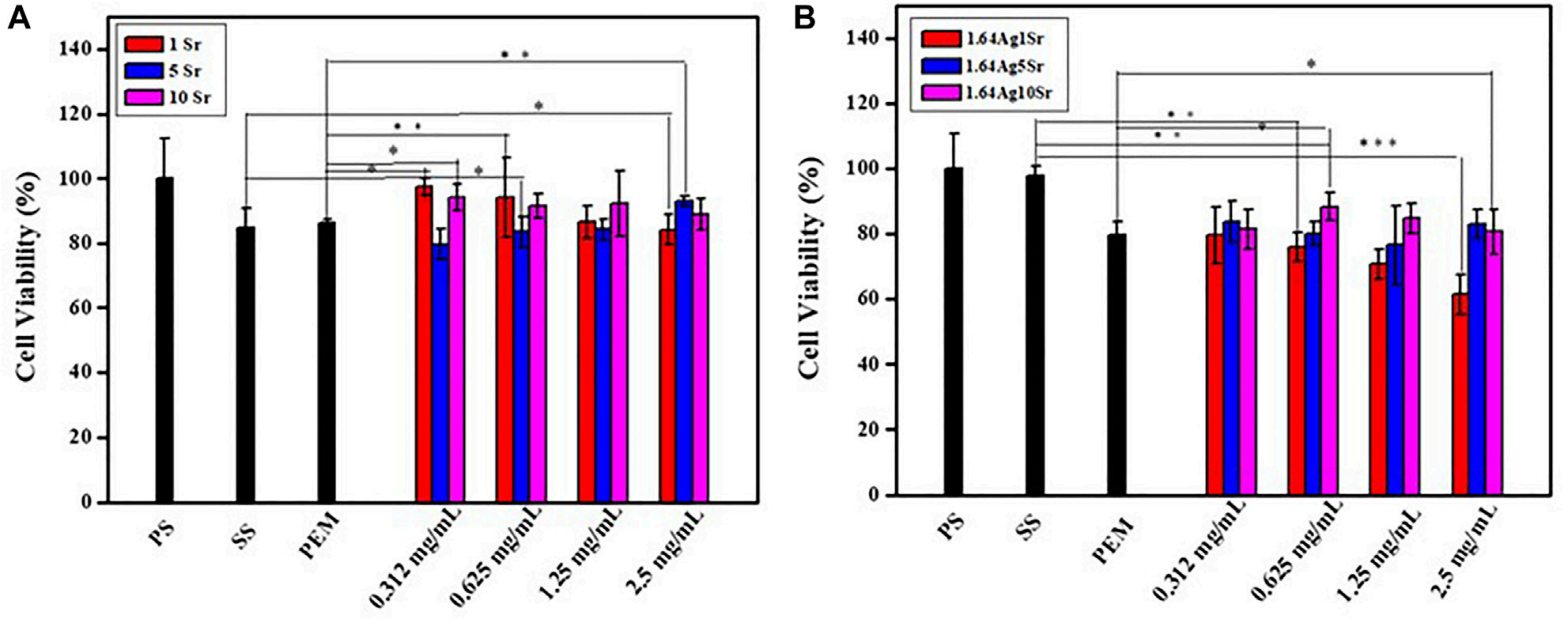
The results of cytotoxicity tests of **(A)** SrMBG and **(B)** AgSrMBG co-cultured with MC3T3-E1 cells. (PS: polystyrene flask) (*, ** and *** indicate *p* value < 0.05, < 0.01 and < 0.001).

The accurate concentrations of the dopants, Ag and Sr ions, in MBG are extremely important to avoid adverse *in vitro* and *in vivo* responses. According to the ICP-OES analysis, the concentrations of SrMBG were 0.971, 4.978, and 8.591 ppm at 1, 5, and 10 mol% of Sr, respectively. The concentrations of AgSrMBG were 1.028, 4.808, and 9.081 ppm at 1, 5, and 10 mol% Sr, respectively. These results suggest well-controlled Ag and Sr concentrations in the AgSrMBG. To determine the appropriate concentrations to be used in the PEM, cytotoxicity tests were performed by coculturing MC3T3-E1 cells with various concentrations of SrMBG and AgSrMBG with (Figure 1). SrMBG showed excellent biocompatibility: cell viability of ∼80% at all concentrations below 0.625 mg/ml (Figure 1A), above which a slight decrease in cell viability was observed. Given the high potential level of 94.06% cell viability, SrMBG (1 mol%) was used in the following experiments. Sr has been reported to promote cell proliferation and angiogenesis, though it lacks antibacterial properties (Liu W. C. et al., 2020). We therefore incorporated Ag as an antibacterial agent in AgSrMBG. The cytotoxicity test results suggest that 1 mol% Ag-containing SrMBG slightly decreased cell viability to 60% at a concentration of 2.5 mg/ml (Figure 1B). AgSrMBG cytotoxicity was significantly increased at 10 mol% Sr. At a 0.625 mg/ml concentration of AgSrMBG, cell viability was lower than 80% at 1 mol% Sr. At the same concentration, the 10 mol% Sr-containing AgSrMBG significantly increased cell viability to 91.64%. Thus, 0.625 mg/ml Ag10SrMBG was selected for PEM preparation. Therefore, these samples performed enhance biological activity towards bone tissue engineering application.

### Cells Viability of Polyelectrolyte Multilayer Films

**FIGURE 2:**
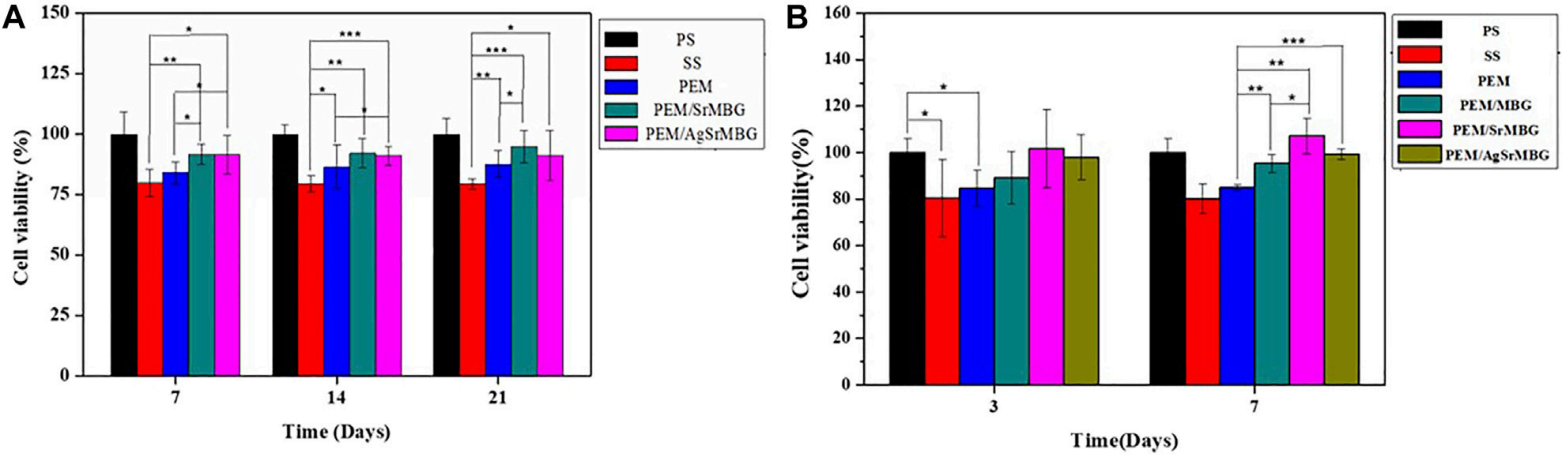
The MTT assay results of samples co-cultured with **(A)** rBMSC for 7, 14, and 21 days, respectively, and **(B)** HUVECs for 3 and 7 days (*, ** and *** indicate *p* value < 0.05, < 0.01 and < 0.001).

The MTT reagents were co-cultured with rBMSCs for 7, 14, and 21 days (Figure 2A) and HUVECs for 3 and 7 days (Figure 2B). SrMBG showed a cell viability of between 91.62 and 107.15% for both cell types after 7 days of co-culturing. After 21 days, cell viability in rBMSCs was still higher than 91.16%. Some cytotoxicity was observed in samples containing Ag due to Ag used as antibiotics which contain slightly toxicity (Pajares-Chamorro et al., 2021) Ag filled SrMBG deposited onto PEM films can promote intense antibacterial properties. However, high cell viability was maintained in AgSrMBG. These results strongly suggest that Sr demonstrates adequate cell viability and regulates the cytotoxicity of Ag. Therefore, these materials possessed excellent biocompatibility as compared to others. Thus, these samples can exhibit intense cell proliferation, differentiation and attachment, bone regeneration that has potential ability for orthopedic application.

### Morphology and Physical Properties of Polyelectrolyte Multilayer Films

**FIGURE 3:**
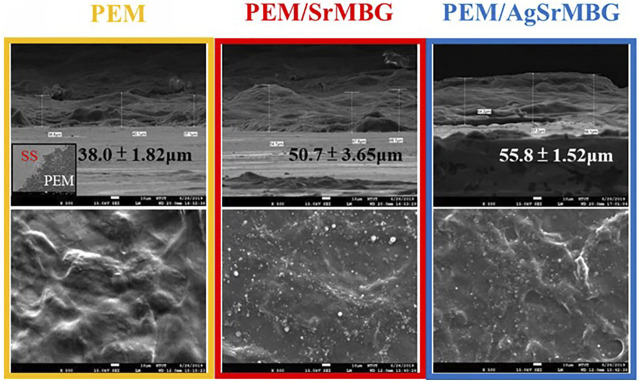
The SEM images of PEM, PEM/SrMBG and PEM/AgSrMBG, respectively (The scale bars represent 10 μm).

The surface properties of the coatings are crucial in determining biological response. SEM imaging shows wrinkled surfaces on the PEM coatings, as seen in Figure 3. The formation of some particles can be identified on the surfaces of the MBG-containing PEM coatings. The thickness of the 20-layers PEM coatings were measured as 38.0 ± 1.82 μm, 50.7 ± 3.65 μm, and 55.8 ± 1.52 μm for PEM, PEM/SrMBG, and PEM/AgSrMBG, respectively. These images suggest that MBG was successfully contained in the PEM coatings. The PEM coating significantly altered the mechanical properties of the SS substrate (Table 1). The surface hardness of SS is 5.64 ± 0.58 GPa and was reduced to 0.34 ± 0.02 GPa after coating with PEM. The surface hardness did not vary significantly compared to PEM when SrMBG and AgSrMBG were added. Young’s modulus of SS decreased from 170.54 ± 5.20 GPa to 6.22 ± 0.26 GPa after PEM coating and showed similar results for the PEM/SrMBG (5.50 ± 1.60 GPa) and PEM/AgSrMBG (5.35 ± 1.55 GPa). Our results are comparable to the hardness and Young’s modulus of bone (0.36–1.2 GPa and 9.64–29.96 GPa) which would promote osteoblast proliferation and attachment (Lee et al., 2013). The initial stage of cell proliferation is based on a suitable environment for cell attachment. Thus, contact angle and surface roughness are crucial properties determining cell attachment (Deligianni et al., 2001). Compared to SS, contact angle was reduced by the PEM coating and surface hydrophilicity was increased by PEM/SrMBG and PEM/AgSrMBG. Thus, PEM/SrMBG and PEM/AgSrMBG samples can intensify the wettability of surface substrate through the deposition onto PEM films (Panda et al., 2021a). Moreover, it is also due to the increase in surface roughness after PEM/SrMBG (371.41 ± 17.20 nm) or PEM/AgSrMBG (374.78 ± 22.27 nm) coatings compared to uncoated SS (15.50 ± 2.25 nm). Therefore, PEM/SrMBG and PEM/AgSrMBG samples can enhance bone regeneration ability, cell proliferation and attchment.

**TABLE 1:** The surface properties of samples.

	Surface hardness (GPa)	Young’s modulus (GPa)	Contact angle (°)	Surface roughness (nm)
SS	5.64 ± 0.58	170.54 ± 5.2	68.63 ± 1.60	15.50 ± 2.25
PEM	0.34 ± 0.02	6.22 ± 0.26	52.29 ± 2.25	204.14 ± 10.27
PEM/SrMBG	0.32 ± 0.08	5.50 ± 1.60	39.68 ± 0.59	371.41 ± 17.20
PEM/AgSrMBG	0.29 ± 0.09	5.35 ± 1.55	37.09 ± 0.88	374.78 ± 22.27

### Cells Proliferation of Polyelectrolyte Multilayer Films

**FIGURE 4:**
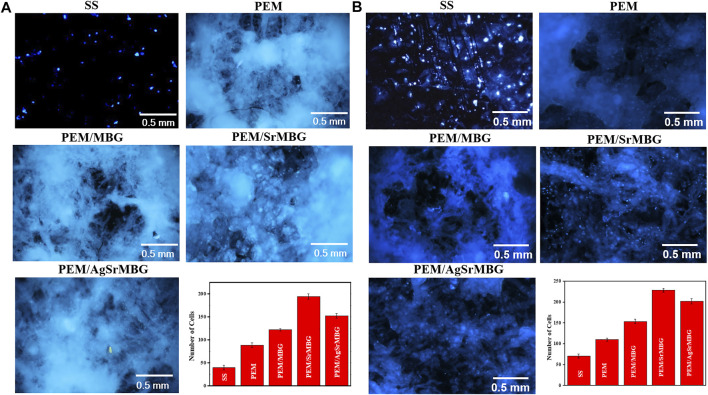
The DAPI fluorescent DNA-stained images for coating samples cocultured with rBMSCs for **(A)** 3 and **(B)** 7 days.

**FIGURE 5:**
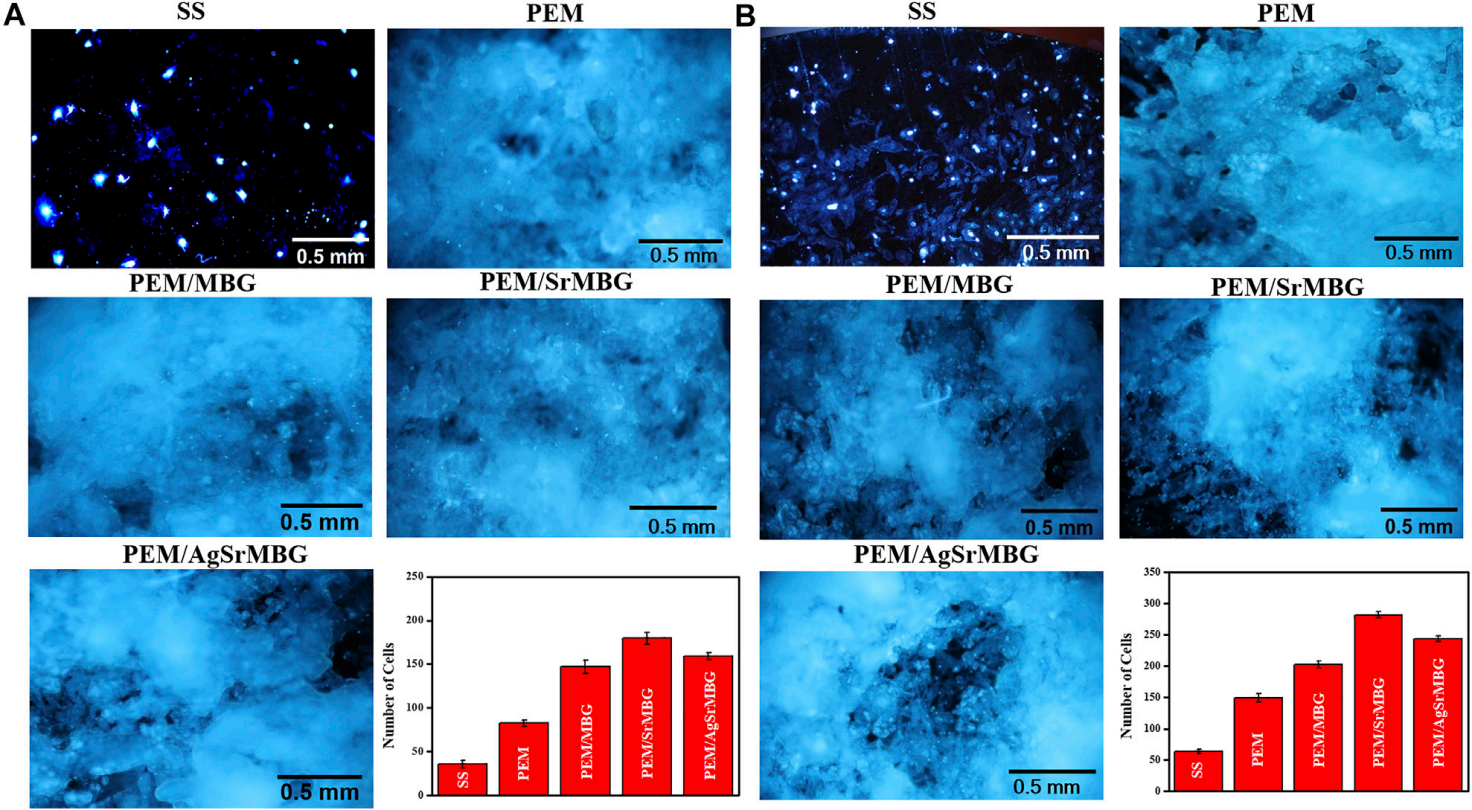
The DAPI fluorescent DNA-stained images for PEM coating samples cocultured with HUVECs for **(A)** 3 and **(B)** 7 days.

Cell attachment and cell proliferation were evaluated by DAPI fluorescent DNA staining, as shown in Figures 4, 5 respectively. The SS substrate had few scattered cells attached to it, even after 7 days of coculturing. All PEM coatings samples were covered by a large number of cells after 3 and 7 days (Figure 4). Cell attachment and proliferation was slightly decreased for the PEM/AgSrMBG sample, likely due to minor cytotoxicity from Ag. Fluorescent staining showed similar cell attachment and cell proliferation rates in HUVECs compared to rBMSCs (Figure 5). Our results indicate that the PEM coating samples have a stronger ability to improve cell attachment and cell proliferation compared with the SS substrate. Although the PEM/AgSrMBG sample has slightly decreased cell proliferation, Sr ion incorporation demonstrates excellent ability to improve cell proliferation under normal and Ag-containing environments. These results are consistent with cytotoxicity results. Therefore, these samples exhibited promoted cell proliferation, differentiation and adhesion properties towards bone tissue engineering application.

### Degradation and AgSr Release of Polyelectrolyte Multilayer Films

**FIGURE 6:**
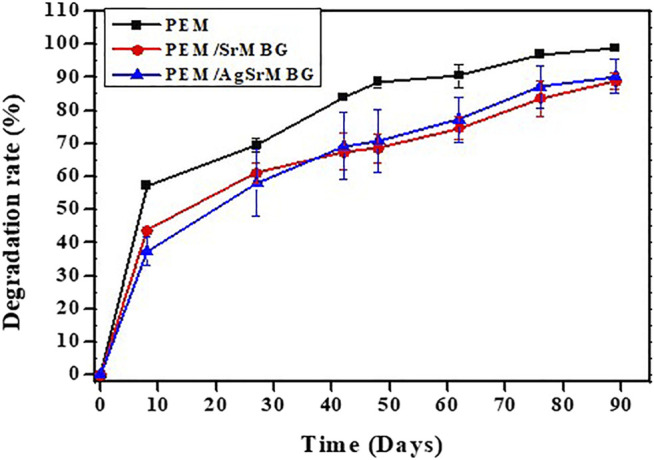
The degradation results of PEM samples treated with PBS for up to 90 days.

**FIGURE 7:**
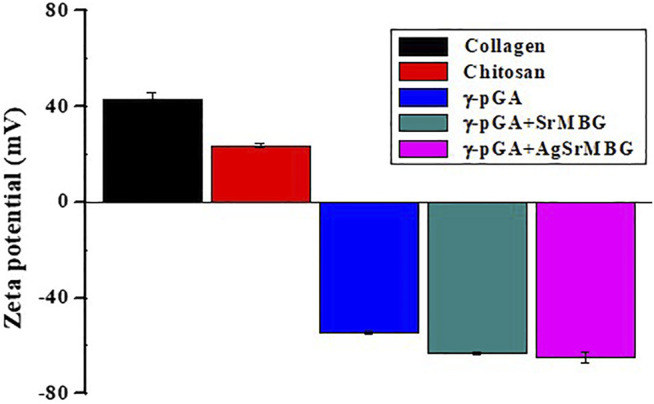
The zeta potential results of PEM coating precursors.

**FIGURE 8:**
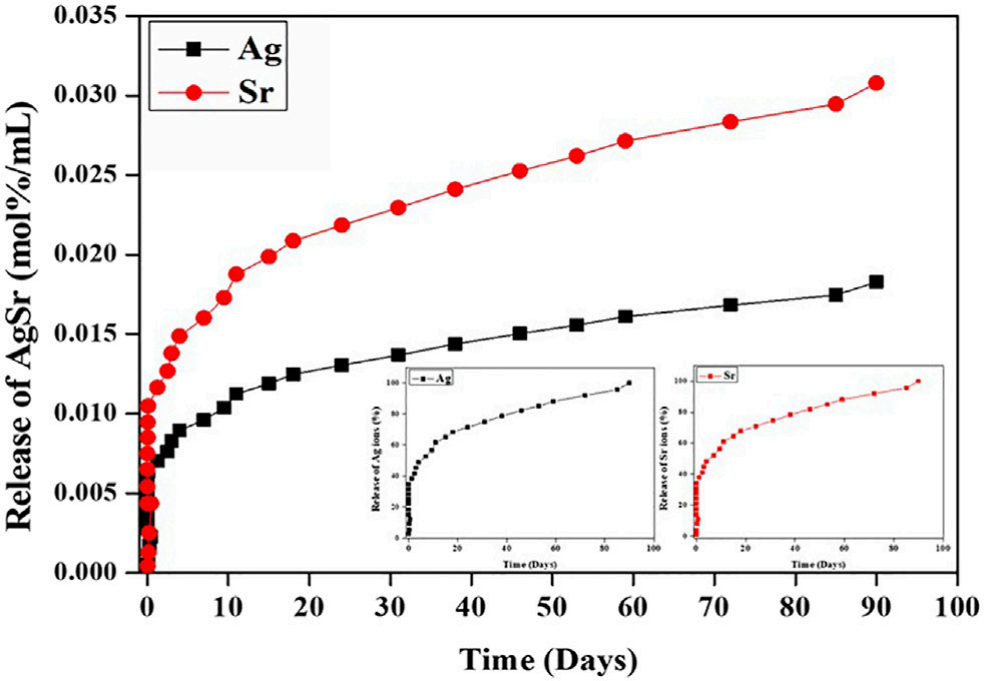
The release rate of Ag and Sr ions from AgSrMBG in PBS across 90 days.

PEM coating has also been regarded as an excellent drug carrier. It can achieve a variety of therapeutic applications by incorporating MBG with antibacterial and angiogenic functions. However, the durability, bioactivity, and antibacterial properties of PEMs need to be verified. The degradation of PEM coatings was performed by soaking them in PBS for up to 90 days and recording daily weight loss (Figure 6). At day eight, PEM coating showed a much greater rate of degradation (>57% of weight lost) compared to PEM/SrMBG (43%) and PEM/AgSrMBG (37%) coatings. All PEM coatings continuously lost weight until the end of the experiment, though the total loss was less in PEM/SrMBG (88%) and PEM/AgSrMBG (90%) coatings compared to the unloaded PEM coating (98%). This results can be attributed to the electrostatic potential of the coating precursors. The zeta potential results demonstrated that SrMBG and AgSrMBG-containing γ-PGA have higher negative charges than pure γ-PGA (Figure 7). The surface potential of each precursor solution significantly affected the final quality of the coating. A large electrostatic potential gap can consequently improve the stability of the coating and decrease degradation rates. As shown in Figure 8, the ion release curves suggest a significant release at the initial stage (<3 days) before stabilization of the material. The subsequent ion release tended to be stable and gradual for up to 90 days.

### Apatite Formation

**FIGURE 9:**
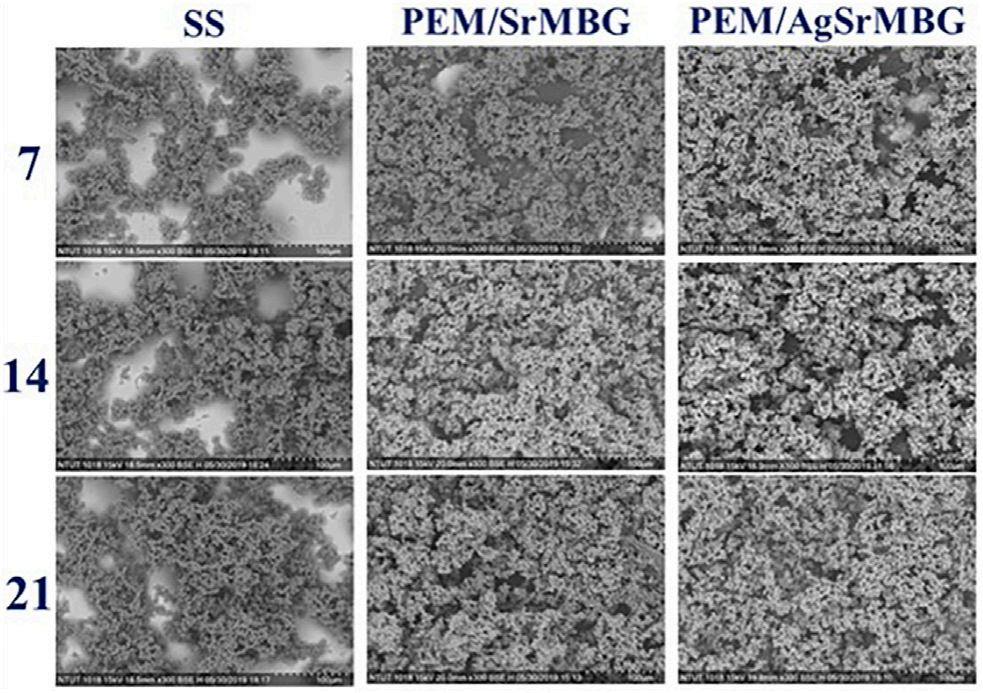
The SEM images show the surface morphologies of SS, PEM/SrMBG, and PEM/AgSrMBG soaked in SBF for 7, 14, and 21 days, respectively.

**FIGURE 10:**
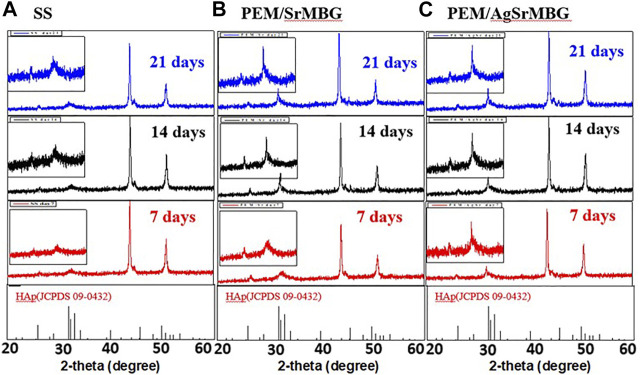
The XRD results of crystal structure on **(A)** SS, **(B)** PEM/SrMBG, and **(C)** PEM/AgSrMBG coating samples soaked in SBF for 7, 14, and 21 days, respectively.

The bioactivities of the PEM coating samples were characterized using SEM and XRD techniques (Figure 9). Samples were soaked in double the normal concentration of SBF to accelerate HAp formation. Sparse HAp formation was observed on the SS surface by day seven, which increased but did not fully cover the surface after 21 days. On the contrary, PEM/SrMBG and PEM/AgSrMBG coatings demonstrated strong bioactivity with thick and dense layers of HAp formed on the surface after 7 days and the surfaces were fully covered by day fourteen (Figure 9). We confirmed the precipitate to be HAp by using XRD to characterize the crystal structure (Figure 10). The XRD results were revealed that PEM/AgSrMBG composite film was shown higher intensity with increased its crystallinity after 21 days SBF soaking as compared to other samples. This XRD matched with JCPDS 09–0,432 of HAp. This result indicates that the hydroxyapatite particles mainly/more number formed in PEM/AgSrMBG films that resulting intensifying bioactivity and osteoconductivity towards orthopaedic application. Moreover, PEM/AgSrMBG films possessed enhance hydrophilicity which can potentially permits more number of HAp grow on its surface (Liu et al., 2021). The PEM/AgSrMBG film performed more intense HAp (211) signal peak after 21 days soaking. Thus, it exhibited more crystalline structure and sharper peaks of apatite layers’ formation on the surface as compared to other samples with days of 7 and 21. Therefore, it revealed enhance bioactivity which can influence towards osteoconductivity.

### Antibacterial Test of Polyelectrolyte Multilayer Films

**FIGURE 11:**
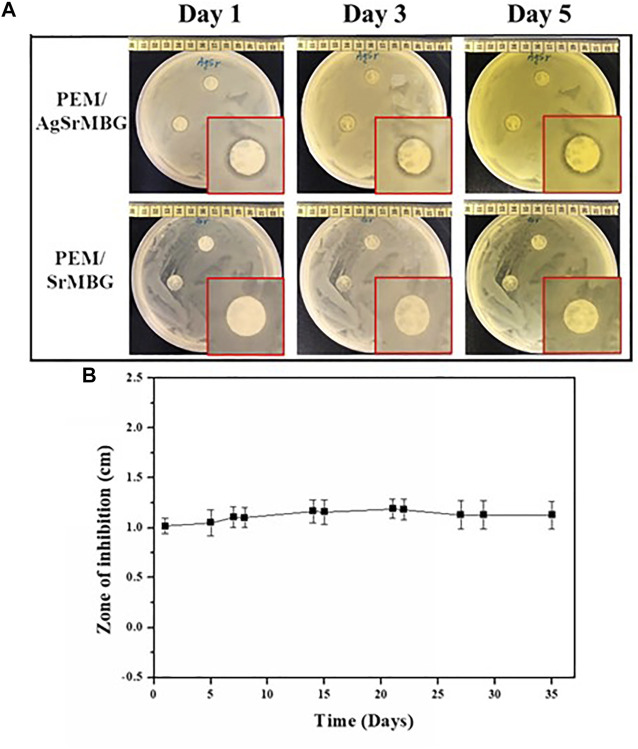
**(A)** Optical images of the antibacterial inhibition zone results for PEM/SrMBG and PEM/AgSrMBG coating samples for 1–3 days, and **(B)** the measured zone of inhibition across 35 days.

To evaluate antibacterial activity, the PEM samples were co-cultured with *E. coli* DH5α and the zone of inhibition was measured for each sample for 35 days (Figure 11). After 3 days, there was a distinct zone of inhibition surrounding the PEM/AgSrMBG samples compared to the PEM/SrMBG samples that did not show obvious inhibition (Figure 11A). Inhibition zone (1 cm) was maintained for more than 1 month in the PEM/AgSrMBG coating, indicating long-term antibacterial properties. AgSrMBG on to the PEM films enhanced the antibacterial properties due to the Ag utilized as intense antibiotic activity (Pajares-Chamorro et al., 2021). These results demonstrate that the PEM/SrMBG and PEM/AgSrMBG coatings can maintain long-term multifunctional drug release, bioactivity, and antibacterial activity. Therefore, these samples can enhance antibacterial activities.

### Alkaline Phosphatase Activity and Mineralization of rBMSCs

**FIGURE 12:**
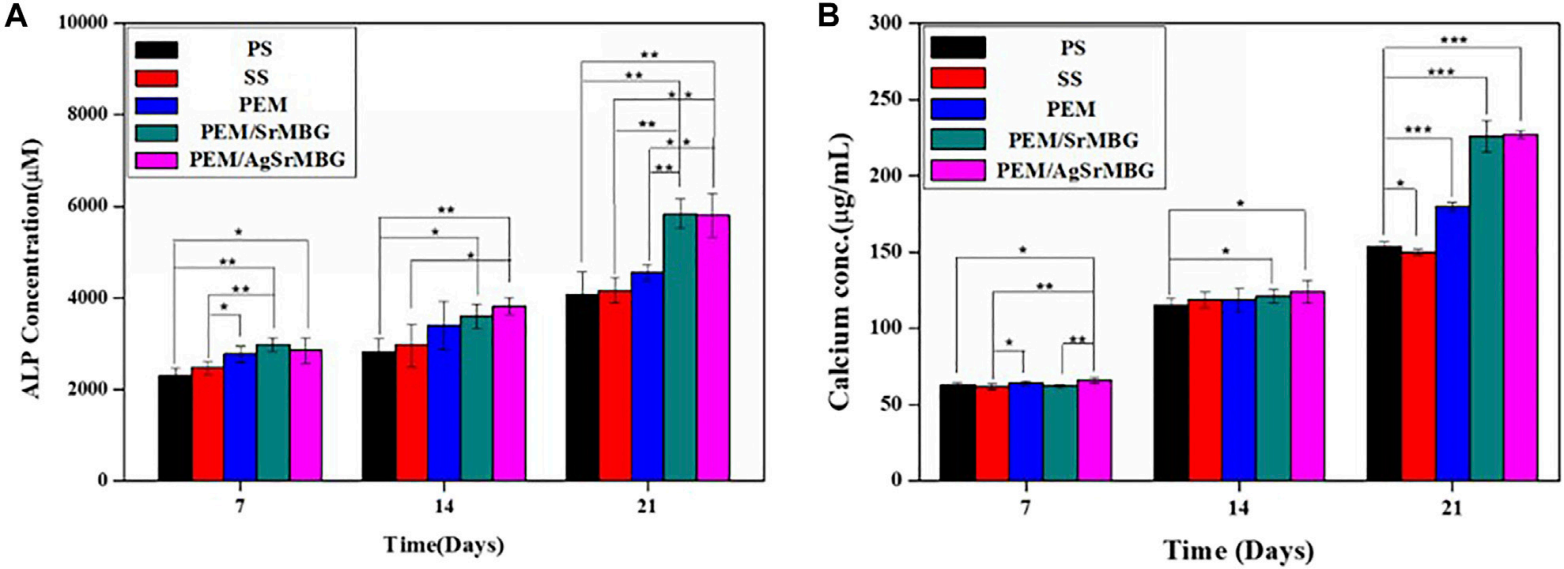
The **(A)** ALP concentrations and **(B)** Ca contents of rBMSCs co-cultured with PS, SS, PEM, PEM/SrMBG and PEM/AgSrMBG samples for 7, 14, and 21 days, respectively. (*, ** and *** indicate *p* value < 0.05, < 0.01 and < 0.001).

ALP and Ca concentrations are key biological indicators of osteogenesis. rBMSCs were cocultured with the different PEM samples for 7, 14, and 21 days (Figure 12). PEM/SrMBG had the highest ALP concentration after 7 days, though PEM/AgSrMBG had a slightly higher ALP concentration at 14 days. At 21 days, PEM/SrMBG and PEM/AgSrMBG samples demonstrated significantly higher ALP concentrations compared to PEM and 316L SS as shown in Figure 12A. Sr-filled with MBG onto the PEM films may enhance osteogenesis and new blood vessels formation (Zhao et al., 2015; Mosaddad et al., 2020). There were no significant differences in Ca content after 7 and 14 days. However, the PEM/SrMBG and PEM/AgSrMBG samples showed higher Ca content after 21 days as presented in Figure 12B. The Sr-inserted MBG onto the PEM films can intensified osteogenic differentiation that occupy increase amount of Ca ontent (Bellucci et al., 2014). Osteogenesis is accelerated by the interaction between ALP and HAp (Mccomb et al., 2013). Osteoblasts secrete a collagen-proteoglycan matrix that binds to Ca salts. Through this binding, the prebone matrix becomes calcified (Gilbert, 2008). Thus, the ALP and Ca contents increased dependently after 14 days of the experiment. Therefore, these materials possessed enhanced angiogenesis, osteogenesis, osteoblastic cell proliferation, differentiation and attachment that has great potential towards orthopedic application.

### Histology Study

**FIGURE 13:**
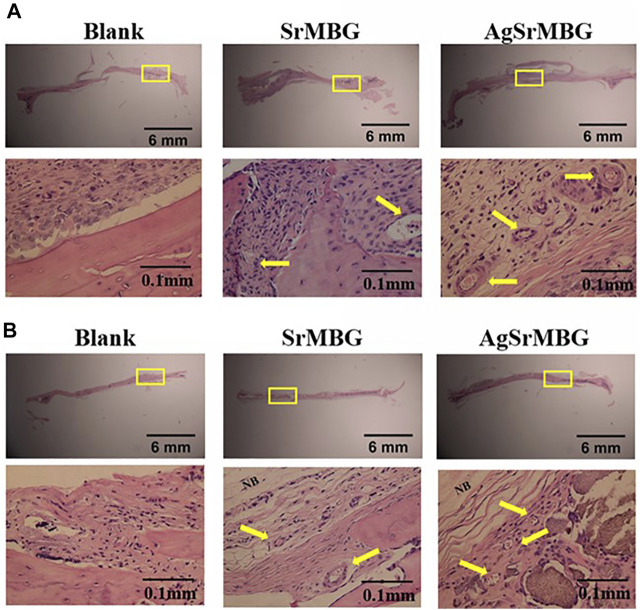
The H&E staining *in vivo* results of packed SrMBG and AgSrMBG discs for **(A)** 1 week and **(B)** 3 weeks after implantation into the skull defects of Sprague-Dawley rats. The yellow square represents the implantation site, yellow arrow is the site of new blood vessel formation, and NB indicates new bone formation. (*n* = 5).

**FIGURE 14:**
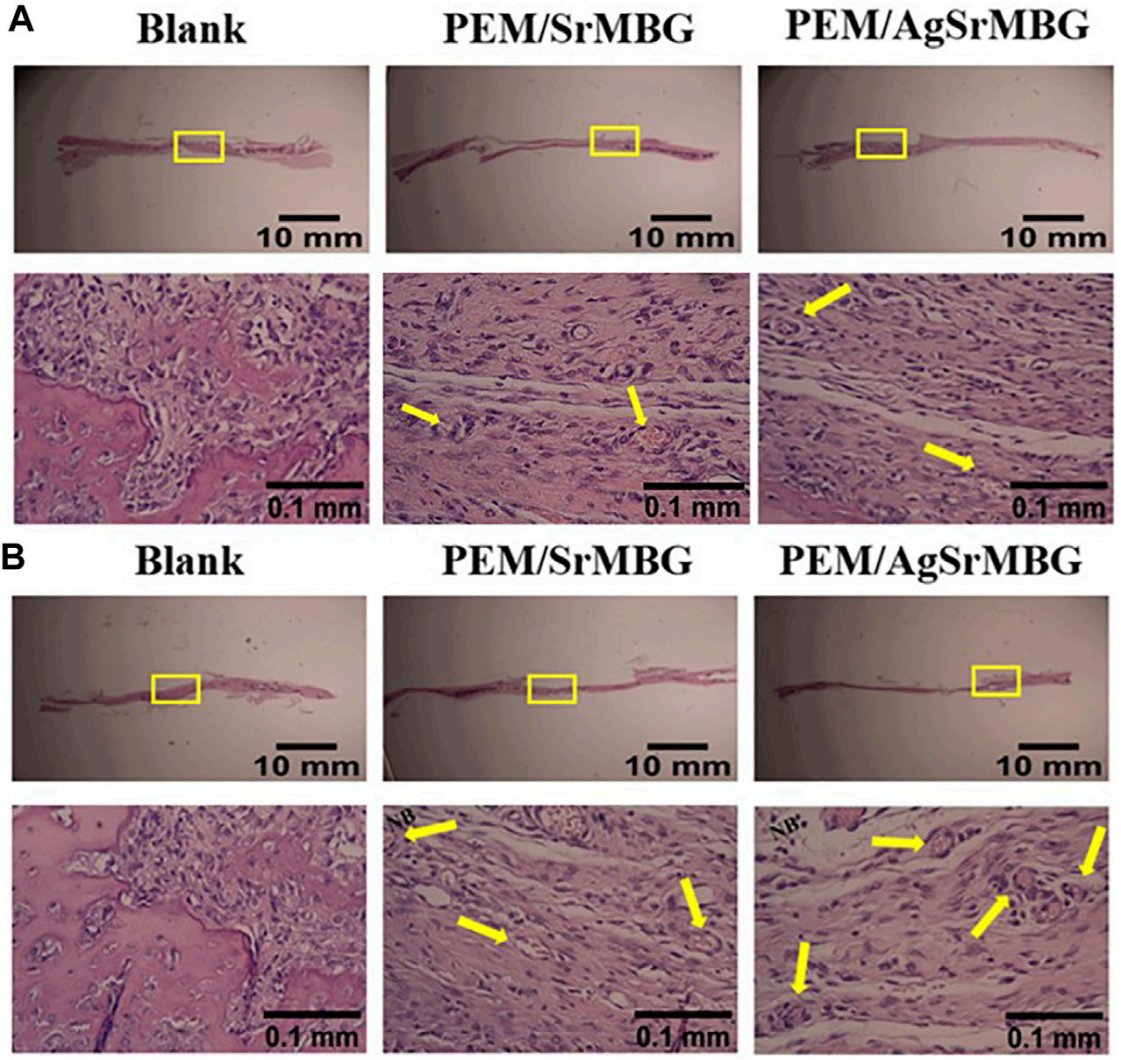
The H&E staining *in vivo* results of PEM/SrMBG and PEM/AgSrMBG **(A)** 1 week and **(B)** 3 weeks of implantation in Sprague-Dawley rat skull defects. The yellow square represents the implantation site, yellow arrows indicate new blood vessel formation sites, and NB represents new bone formation. (*n* = 5).

In order to observe the biological responses *in vivo* of SrMBG and AgSrMBG samples, they were implanted into the skull defect of Sprague-Dawley rats. A thin layer of fibrous tissue was developed on the surface of samples after 1 and 3 weeks. As shown in Figure 13, the SrMBG and AgSrMBG samples showed a stronger angiogenic ability after 1 week compared to the control. After 3 weeks, some new bone formation was observed in both SrMBG and AgSrMBG samples. The results convinced the enhanced biocompatibility of SrMBG and AgSrMBG. Further implantation results for PEM/SrMBG and PEM/AgSrMBG are shown in Figure 14. SrMBG and AgSrMBG containing PEM coatings exhibited intense blood vessels after 3 weeks as compared to 1 week. These PEM/SrMBG and PEM/AgSrMBG samples blood vessel density was increased significantly after 3 weeks than that of 1 week. The blood distribution occurs in the granulated tissue cavities which influence for new blood vessel formation. The formation of new blood vessel plays an important role in infracted tissues of survival cells. So, the density of blood vessel was considered within that region, after 7 and 21 days’ treatment of samples analyzation (Gao et al., 2020). Moreover, PEM/SrMBG and PEM/AgSrMBG possessed new bone generation over the time periods. Sr filled porous BG may enhance bioactivity of the bone regeneration and new blood vessels formation (Marie et al., 2011; Wu et al., 2015; Zhao et al., 2015; Liu et al., 2016). Furthermore, the PEM/SrMBG and PEM/AgSrMBG films performed intensified angiogenesis and osteogenic behaviour that was well agreed with ALP and Ca content results. Therefore, from the above the histological (H&E) staining results after 3 weeks revealed that PEM/SrMBG and PEM/AgSrMBG films can promote angiogenesis, osseointegration and osteogenesis. This material demonstrates long-term antibacterial, angiogenic, and osseointegrative abilities maintained by long-term Ag and Sr ion release. Hence, these materials consider as great potential towards orthopedic applications.

## Conclusion

SrMBG and AgSrMBG-containing PEM coatings can sustain long-term antibacterial, angiogenic, and osseointegrative activities. These findings are based on sustained Ag and Sr ion release for up to 90 days, inhibition zone tests for 35 days, and *in vivo* testing for 21 days. Even Ag demonstrated slight cytotoxicity *in vitro*, the incorporation of Sr improved cell viability and reduced the cytotoxicity of Ag. The histological (H&E) staining images suggest new blood vessel formation after 1 week and new bone formation after 3 weeks. These results are comparable to the *in vitro* ALP concentration and Ca content analyses. SrMBG and AgSrMBG-containing PEM coatings have demonstrated to be promising materials for effective osseointegrated implants.

## Data Availability

The original contributions presented in the study are included in the article/Supplementary Material, further inquiries can be directed to the corresponding author.
